# PRKCQ inhibition enhances chemosensitivity of triple-negative breast cancer by regulating Bim

**DOI:** 10.1186/s13058-020-01302-w

**Published:** 2020-06-29

**Authors:** Jessica H. Byerly, Elisa R. Port, Hanna Y. Irie

**Affiliations:** 1 Division of Hematology and Medical Oncology, Department of Medicine, New York, USA; 2 Department of Surgery, Mount Sinai Hospital, New York, NY 10029 USA; 3 Department of Oncological Sciences, Tisch Cancer Institute, Icahn School of Medicine at Mount Sinai, 1468 Madison Avenue, New York, NY 10029 USA

**Keywords:** PRKCQ, Bim, Apoptosis, Triple-negative breast cancer

## Abstract

**Background:**

Protein kinase C theta, (PRKCQ/PKCθ) is a serine/threonine kinase that is highly expressed in a subset of triple-negative breast cancers (TNBC) and promotes their growth, anoikis resistance, epithelial-mesenchymal transition (EMT), and invasion. Here, we show that PRKCQ regulates the sensitivity of TNBC cells to apoptosis triggered by standard-of-care chemotherapy by regulating levels of pro-apoptotic Bim.

**Methods:**

To determine the effects of PRKCQ expression on chemotherapy-induced apoptosis, shRNA and cDNA vectors were used to modulate the PRKCQ expression in MCF-10A breast epithelial cells or triple-negative breast cancer cells (MDA-MB231Luc, HCC1806). A novel PRKCQ small-molecule inhibitor, 17k, was used to inhibit kinase activity. Viability and apoptosis of cells treated with PRKCQ cDNA/shRNA/inhibitor +/-chemotherapy were measured. Expression levels of Bcl2 family members were assessed.

**Results:**

Enhanced expression of PRKCQ is sufficient to suppress apoptosis triggered by paclitaxel or doxorubicin treatment. Downregulation of PRKCQ also enhanced the apoptosis of chemotherapy-treated TNBC cells. Regulation of chemotherapy sensitivity by PRKCQ mechanistically occurs via regulation of levels of Bim, a pro-apoptotic Bcl2 family member; suppression of Bim prevents the enhanced apoptosis observed with combined PRKCQ downregulation and chemotherapy treatment. Regulation of Bim and chemotherapy sensitivity is significantly dependent on PRKCQ kinase activity; overexpression of a catalytically inactive PRKCQ does not suppress Bim or chemotherapy-associated apoptosis. Furthermore, PRKCQ kinase inhibitor treatment suppressed growth, increased anoikis and Bim expression, and enhanced apoptosis of chemotherapy-treated TNBC cells, phenocopying the effects of PRKCQ downregulation.

**Conclusions:**

These studies support PRKCQ inhibition as an attractive therapeutic strategy and complement to chemotherapy to inhibit the growth and survival of TNBC cells.

## Background

Triple-negative breast cancer (TNBC) represents approximately 15% of all diagnosed breast cancer cases. Chemotherapy remains the main systemic treatment for these cancers, with few biologic or targeted therapy options, and sensitivity of a patient’s TNBC to chemotherapy is a strong predictor of long-term outcomes [1, 2]. Although many patients do well with standard-of-care chemotherapy, some TNBCs have incomplete responses leading to metastatic recurrences that are associated with significant morbidity and mortality. Therefore, new therapeutic strategies that suppress the growth and spread of TNBC cells could improve outcomes and quality of life for patients.

PRKCQ/PKCθ is a serine/threonine kinase that is a member of the novel PKC family (reviewed in [3, 4]). We first identified PRKCQ as a candidate regulator of anchorage-independent survival of breast cancer cells in a functional kinome screen [5]. PRKCQ is characterized by a unique protein domain structure consisting of diacylglycerol binding sites, but lacking Ca+ binding sites typical of classical PKCs. PRKCQ maps to chromosome 10p15, a region frequently mutated in T cell leukemia, lymphomas, and T cell immunodeficiencies [3].

PRKCQ is widely expressed throughout the hematopoietic system, primarily in T cells, mast cells, NK cells, and platelets, as well as in the skeletal muscle, liver, thymus, and the nervous system [3]. Much of the known isoform-specific functions of PRKCQ are in the context of immune function; mice deficient in PRKCQ expression exhibit defects in T cell activation due to impaired Ca^+ 2^ signaling, NFAT, and NFkB activation [6–8]. PRKCQ also regulates the survival of T cells by regulating the expression of pro- and anti-apoptotic Bcl2 family members [9]. Evidence supports a relatively specific role for PRKCQ in immune responses; it is dispensable for much of immunity against viral and bacterial pathogens [10]. In contrast, PRKCQ appears to be required for immune responses associated with autoimmune diseases and allograft rejection, perhaps due to a specific requirement for PRKCQ in the maturation of Th17 cells, a subset of CD4^+^ T cells [11, 12].

In contrast, the function of PRKCQ in non-hematopoietic tissues and cancers remains to be fully elucidated. PRKCQ is expressed in a subset of GIST cancers and regulates their proliferation [13]. We previously reported that PRKCQ is sufficient to drive growth factor-independent proliferation, migration, and survival of breast epithelial cells by activating Erk/MAPK activity in a kinase activity-dependent manner [14]. PRKCQ protein is not only sufficient to drive these phenotypes but is required for growth in vitro and in vivo of triple-negative breast cancer cells in which it is expressed. PRKCQ expression directly suppresses the expression of ERα in breast cancer cells and is required for c-rel-induced mammary tumorigenesis [15]. PRKCQ also stimulates breast cancer cell migration by stabilizing the expression of Fra-1 in TNBC cells [16]. Finally, PRKCQ also promotes epithelial-mesenchymal transition of breast cancer cells by direct phosphorylation and activation of LSD1, which provides another mechanism by which PRKCQ promotes growth and spread of breast cancer [17].

Here, we have identified a critical role for PRKCQ in the regulation of chemotherapy sensitivity by a novel mechanism; PRKCQ regulates basal levels of Bim, a pro-apoptotic Bcl2 family member that serves as a set point molecule for sensitivity to chemotherapy and targeted therapies. Our studies with a novel PRKCQ kinase inhibitor also highlight the importance of kinase activity in the regulation of Bim and chemotherapy sensitivity. These results support PRKCQ as an attractive therapeutic target for TNBC with clinical translation potential.

## Methods

### Reagents, cells, and cell culture

#### Cells

MDA-MB-231-luc-D3H2LN (referred to as MDA-231-luc) cells were originally obtained from PerkinElmer. Cells were cultured in MEM with Earle’s Salts supplemented with non-essential amino acids, GlutaMAX™, sodium pyruvate, penicillin/streptomycin (Life Technologies), and 10% heat-inactivated fetal bovine serum (Life Technologies) at 37 °C in 5% CO_2_. MCF10A cells were obtained from ATCC and cultured in 1:1 DMEM/F12 supplemented with 5% horse serum, 20 ng/mL EGF (PeproTech), 500μg/mL hydrocortisone (Sigma-Aldrich), 100 ng/mL cholera toxin (Sigma-Aldrich), 10μg/mL insulin (Sigma-Aldrich), and penicillin/streptomycin (Life Technologies). HCC1806 cells were purchased from ATCC and cultured in RPMI supplemented with 10% heat-inactivated fetal bovine serum (Life Technologies) and 1% penicillin/streptomycin (Life Technologies). MDA-MB157 cells were obtained from ATCC and cultured according to ATCC instructions.

#### Inhibitors and chemotherapy agents

Paclitaxel from *Taxus yannanensis* and doxorubicin hydrochloride were purchased from Sigma. Z-VAD-FMK was purchased from APExBIO. 17k was obtained from Abbvie, and its structure is described in [18].

#### Antibodies

Antibodies directed against the following proteins were obtained from the indicated suppliers: AbCam—rabbit monoclonal protein kinase C (PKC-θ) [EPR1487(2)]; BD Biosciences—Vimentin (RV202); Invitrogen—phospho-PKCθ (Thr538) antibody (F4H4L1), ABfinity™; Cell Signaling Technologies—PKCδ (D10E2), PKCα, PKD/PKCμ (D4J1N) phospho-PKCθ (T538), BIM (C34C5), Vimentin (D21H3) XP, MCL-1 (D35A5), BCL-2 (124), cleaved PARP (Asp214), Survivin (71G4B7), BCL-XL (54H6), β-tubulin (9F3), integrin β5; Santa Cruz biotechnology—GAPDH (G9) mouse monoclonal secondary antibodies conjugated with HRP and directed against either rabbit or mouse were purchased from Cell Signaling.

### Western blot analysis

Cells were lysed in NP40 lysis buffer (Invitrogen™) containing 1% Halt Protease Inhibitor Cocktail (Thermo Scientific) and 10% PhosSTOP (Sigma-Aldrich). Lysates were cleared and stored at − 80 °C. Protein was quantified using the Pierce™ BCA Protein Assay Kit (Thermo Scientific™). Samples were prepared using Laemmlli buffer (SDS—Sample Buffer, Reducing, 4x; Boston Bioproducts). At least 15 μg of protein per sample was resolved on NuPAGE™ Bis-Tris 4–12% gels (Invitrogen) in NuPage™ MOPS SDS Running Buffer (Invitrogen™). Protein was transferred to the PVDF membrane and blocked in either 3–5% bovine serum albumin (BSA), protease-free (Roche), or non-fat dry milk (NFDM; Cell Signaling). Blots were developed using Pierce ECL Western Blotting Substrate (Thermo Scientific™^)^, Immobilon Crescendo Western HRP substrate (EMD Millipore), or Immobilon Forte Western HRP substrate (EMD Millipore).

### Constructs, viral production, and stable cell line generation

Constructs encoding short hairpin RNA sequences targeting PRKCQ (TRCN0000001791, TRCN0000199654, and TRCN0000197216 referred to as 91, 54, and 16, respectively) were purchased from Open Biosystems/Thermo Scientific. Bim shRNAs (TRCN0000001054 (54) and TRCN0000356026 (26)) were purchased from Sigma Aldrich (St. Louis, MO, USA). Viral packaging 293T cells were transfected according to standard protocols to produce lentiviral particles. Viral supernatant was collected 24, 48, 72, and 96 h post-transfection and pooled. HCC1806 and MDA-231-luc cells were infected in the presence of 2μg/mL polybrene (Sigma -Aldrich). Cells were exposed to viral supernatant overnight before changing to complete media. PRKCQ mutants (A148E and K409R) were generated as previously described [14, 19].

### siRNA reagents and transfection

#### Reagents

Invitrogen™: Oligofectamine™ transfection reagent; Horizon Discovery: siGENOME Human BCL2L11 (Bim), SmartPool; siGENOME Non-Targeting siRNA #5. Cells were transfected according to the manufacturer’s protocol for Oligofectamine™ transfection reagent in a 6-well culture plate. Briefly, siRNAs directed against Bim or a non-targeted control were diluted in serum-free medium. In separate tubes, Oligofectamine™, 3 μL/sample, was diluted in serum-free medium. The siRNA-containing medium and the Oligofectamine™-containing medium were mixed and incubated at room temperature for 15 min for complexes to form. Following incubation, 200 μL of the siRNA/Oligofectamine™ mixture was carefully pipetted into each well of cells, already containing 800 μL of serum-free medium. This resulted in a final siRNA concentration of 50 nM. Cells were incubated for 3–4 h before the addition of complete cell culture medium containing 30% serum.

### Anoikis and cell death assays

Anoikis (anchorage-independent viability) assay was performed by culturing cells in suspension on polyhema-coated (Sigma-Aldrich) plates, harvested after 24 or 48 h, and cell death assessed using the Cell Death ELISA Kit (Roche) according to the manufacturer’s instructions.

### Cell viability/ apoptosis assays

Cell viability was assessed using either alamarBlue™ (Invitrogen™), CellTiter-Glo® Luminescence Cell Viability Assay (Promega), or CellTiter-Glo® 3D Luminescence Cell Viability Assay (Promega), according to the manufacturer’s protocols. Caspase activation was measured using the Caspase Glo® 3/7 Assay System (Promega).

### 3D culture

Three-dimensional (3D) Matrigel™ (BD Biosciences) cultures were performed as described previously [20].

### Matrigel Transwell invasion assays

Twenty-four-well inserts with 8-μm pores and pre-coated with Matrigel were purchased from Corning. MDA-MB231Luc cells were plated in triplicate (1 × 10^4^ cells/well) in serum-free media with DMSO or 1 μM 17k (inner chamber). Media containing serum was placed in the outer chamber. Cells were allowed to invade for 12 h. Inserts were fixed, washed, and stained with crystal violet. Invaded cells/Transwell were counted.

### Quantitative PCR

RNA was isolated using Qiagen RNEasy. Synthesis of cDNA was carried out using the TaqMan™ Reverse Transcription Reagents Kit (Invitrogen™). Assays for BIM (Hs00708019_s1, Invitrogen™), GAPDH (Hs02758991_g1, Invitrogen™), and β-2 microglobulin (Hs00984230_m1, Invitrogen™) were performed according to the manufacturer’s instructions. Alternatively, qRT-PCR was performed using the following primers as previously described [21]: BIM-EL, forward 5′-GTG GGT ATT TCT CTT TTG ACA CAG AC-3′; BIM-L, forward, 5′-TAC AGA CAG AGC CAC AAG ACA G-3′; and common reverse primer, 5′-GTT CAG CCT GCC TCA TGG AAG-3′; and RPS17 Quantitect primers (QIAGEN). In this case, 2x-All-in-One™ qPCR mix (Genecopoeia) was used according to the manufacturer’s instructions. All qPCR was performed using a Biorad CFx96 thermal cycler.

### Cell cycle analysis/flow cytometry

#### Annexin V staining

Cells were plated at a density of 1 × 10^5^ cells per well in a 6-well plate. Twenty-four hours later, cells were treated with DMSO or paclitaxel (Sigma) at final concentrations of 25 nM and 100 nM. Cells were incubated for 15 h, harvested with 0.05% Trypsin/EDTA (Life Technologies), and washed 1 time with 1× PBS. Samples were brought up in 250 μL of 1× staining buffer containing 5 μL of FITC and 5 μL of PI (FITC/Annexin V Apoptosis Detection Kit I BD Biosciences). Samples were protected from the light and incubated at room temperature for 15 min. An additional 250 μL of 1× staining buffer was added to each sample prior to filtering the sample through 35-μM filter capped tubes (Corning™ Falcon™). Annexin-positive cells were assessed using BD Fortessa and analyzed using the FACS DIVA or FCS express 6 software.

### Statistical calculations

Significance was calculated using standard Student’s *t* test unless indicated otherwise in the figure legends.

## Results

### Kinase-active PRKCQ suppresses chemotherapy-induced apoptosis

**Fig. 1:**
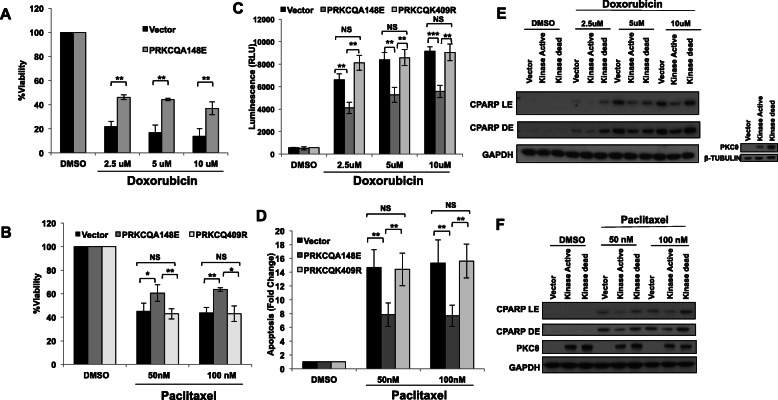
PRKCQ is sufficient to promote resistance to chemotherapy. Overexpression of kinase-active PRKCQ enhances cell viability in the presence of doxorubicin (**a**) or paclitaxel (**b**). Percent viability relative to vehicle control was determined using alamarBlue™. Kinase-active PRKCQ overexpression decreases apoptosis in response to doxorubicin or paclitaxel as determined by Caspase Glo® 3/7 Assay (**c**, **d**) and levels of cleaved PARP (CPARP) (**e**, **f**). Western blots are representative of at least 3 independent experiments. LE, light exposure; DE, dark exposure. All significance was determined using Student’s *t* test except **b**. Here, significance was calculated using Welch’s *t* test. PRKCQA148E: kinase active PRKCQ; PRKCQK409R: catalytically inactive PRKCQ

Enhanced PRKCQ expression is sufficient to promote growth factor-independent growth, anoikis resistance, and migration of breast epithelial cells (MCF-10A) (Byerly et al. [14]). PRKCQ overexpression is also sufficient to enhance the viability of these cells in the presence of chemotherapeutic agents commonly used in the treatment of patient triple-negative breast cancers, such as doxorubicin and paclitaxel (Fig. 1a, b). The increased viability is due to the suppression of chemotherapy-induced apoptosis, as assessed by decreased levels of caspase activity (as detected by Caspase Glo® 3/7 Assay) (Fig. 1c, d) and decreased levels of cleaved PARP (Fig. 1e, f). The suppression of chemotherapy-induced apoptosis by PRKCQ is dependent on the catalytic activity of PRKCQ, as overexpression of kinase-inactive PRKCQ failed to suppress apoptosis in paclitaxel or doxorubicin-treated cells (Fig. 1c–f).

### PRKCQ downregulation enhances chemotherapy-induced apoptosis of TNBC cells

**Fig. 2:**
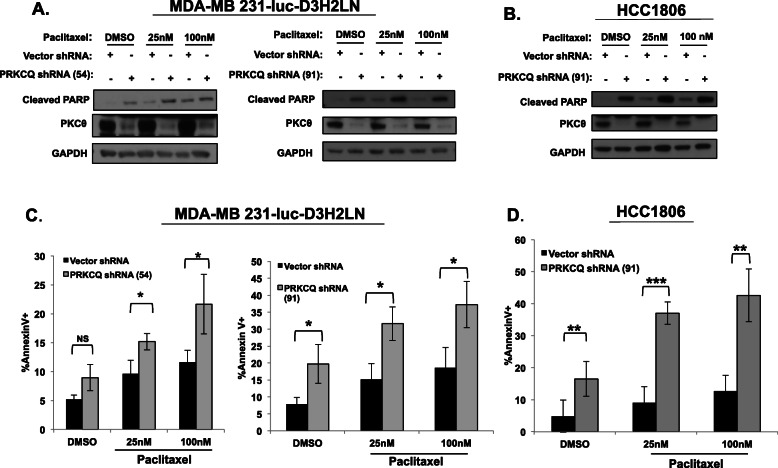
PRKCQ downregulation enhances chemotherapy-induced apoptosis of TNBC cells. MDA-MB231Luc cells (**a**, **c**) or HCC1806 cells (**b**, **d**) infected with control or PRKCQ shRNA-expressing lentivirus were treated with vehicle or paclitaxel chemotherapy for 24 h. Apoptosis was assessed by Western (levels of cleaved PARP) (**a**, **b**) or flow cytometry analysis for Annexin V+ cells (**c**, **d**). Western blots are representative of at least 3 independent experiments. Annexin V+ experiments are averages of at least 3 independent experiments. Significance was determined using Student’s *t* test

As PRKCQ is sufficient to promote resistance to chemotherapy treatment, we sought to determine whether PRKCQ inhibition enhances the sensitivity of TNBC cells to chemotherapy treatment. Downregulation of PRKCQ expression using two independent shRNA vectors increased basal and paclitaxel-associated apoptosis of MDA-MB231Luc and HCC1806 cells, with increased detection of cleaved PARP and increased levels of Annexin V+ cells (Fig. 2a–d). These results support benefits for combined PRKCQ inhibition and chemotherapy in suppressing TNBC viability.

### PRKCQ regulates chemotherapy sensitivity by modulating Bim expression

Given our findings that PRKCQ levels impact the sensitivity of TNBC cells to the apoptotic effects of paclitaxel chemotherapy, we sought to determine the mechanism responsible for this regulation. The balance between pro- and anti-apoptotic Bcl2 family members often determines whether apoptosis is triggered by chemotherapy or targeted therapies [22]. For example, lower basal expression levels of Bim, a pro-apoptotic member of the Bcl2 family, have been associated with relative resistance to chemotherapy and targeted therapies for several tumor types [23–25].

**Fig. 3:**
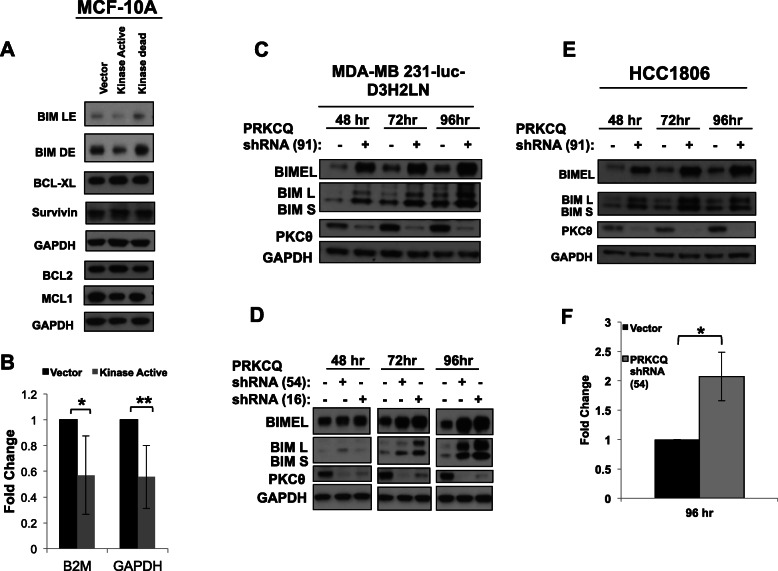
PRKCQ regulates chemotherapy sensitivity by modulating Bim expression. **a** Basal levels of Bcl2 family proteins were assessed in immortalized MCF-10A breast epithelial cells overexpressing vector control or PRKCQ (kinase-active or kinase-dead). **b** Levels of Bim transcript were assessed in MCF-10A breast epithelial cells overexpressing vector control or kinase-active PRKCQ by TaqMan assay. Fold change in Bim transcript levels was calculated with normalization to either of two control housekeeping transcripts (Beta-2 microglobulin or GAPDH). **c**–**e** Bim protein expression in MDA-MB 231-luc and HCC1806 cells was assessed 48 h, 72 h, and 96 h post-infection with PRKCQ shRNA-expressing lentivirus. **f** Levels of Bim EL transcript were determined in MDA-231Luc cells expressing control or PRKCQ shRNA 54 using Bim EL-specific primers, as indicated in the “Methods” section

We evaluated the effect of PRKCQ overexpression or downregulation on the basal expression of pro- and anti-apoptotic Bcl2 proteins. Overexpression of a catalytically active but not kinase-dead PRKCQ suppressed basal expression of pro-apoptotic Bim without significantly altering the expression of other Bcl2 proteins like Mcl1 and Bcl2 (Fig. 3a). This suppression of Bim protein is at least partially due to the suppression of Bim transcript levels (Fig. 3b). Downregulation of PRKCQ in MDA-MB231-Luc and HCC1806 TNBC cells increased basal expression of Bim protein and transcript levels (Fig. 3c–f).

**Fig. 4:**
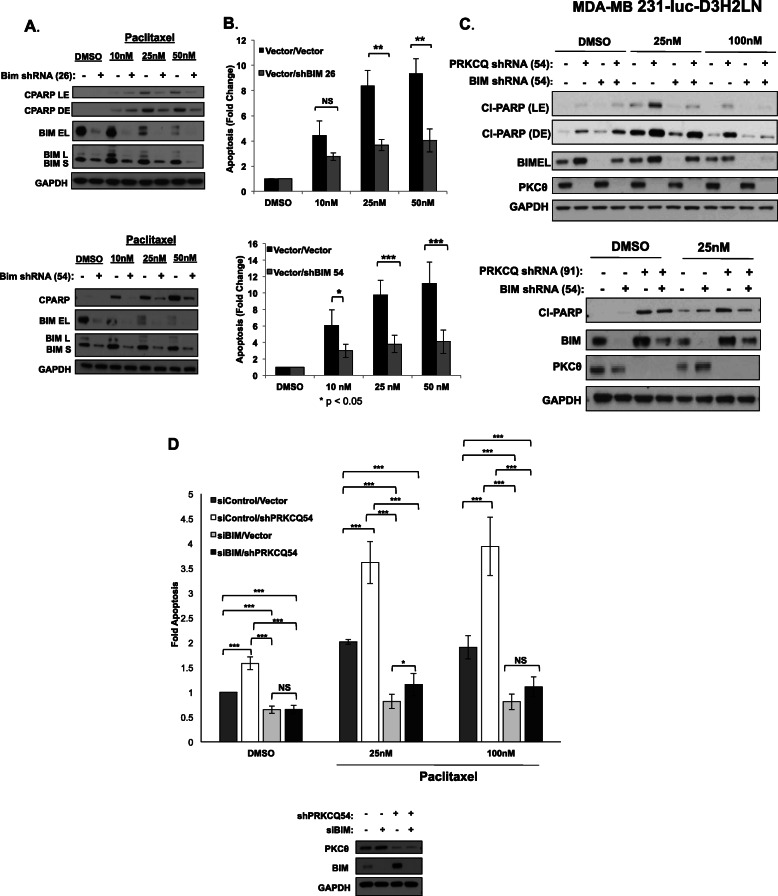
Bim levels determine chemotherapy sensitivity. Levels of apoptosis were assessed following 24 h paclitaxel treatment of MCF-10A cells expressing control or two different Bim shRNA vectors by Western analysis for cleaved PARP (**a**) or Caspase Glo® 3/7 Assay (**b**). **c** Levels of cleaved PARP were assessed in MDA-MB231 Luc cells co-expressing PRKCQ and Bim shRNA following 24 h treatment with vehicle control or paclitaxel. Western blots are representative of at least 3 independent experiments. **d** Apoptosis in MDA-MD231 Luc cells expressing PRKCQ shRNA +/-Bim siRNA and treated with DMSO or paclitaxel was assessed using Caspase Glo® 3/7 Assay. Fold change in apoptosis relative to vector/siRNA control-expressing cells treated with DMSO is shown. Results are averages of at least 3 independent experiments. Significance was determined using Student’s *t* test. NS, not significant. *p < 0.05; **p < 0.01; ***p < 0.001. LE, light exposure; DE, dark exposure

To determine whether modulation of Bim expression is sufficient to impact chemotherapy sensitivity, we evaluated apoptosis in cells expressing control or two different Bim shRNA vectors. Bim downregulation in immortalized, non-transformed MCF-10A cells was sufficient to suppress apoptosis of paclitaxel-treated cells, with decreased levels of cleaved PARP and Annexin V+ cells (Fig. 4a, b).

The increase in Bim caused by PRKCQ downregulation is also required for the synergism between PRKCQ shRNA and paclitaxel chemotherapy in inducing apoptosis. Co-expression of Bim shRNA suppressed apoptosis of paclitaxel-treated, PRKCQ shRNA-expressing TNBC cells, as determined by levels of cleaved PARP and caspase activity (Fig. 4c, d). These data support Bim regulation in PRKCQ-dependent regulation of sensitivity to paclitaxel treatment.

### A novel PRKCQ small-molecule kinase inhibitor suppresses viability and enhances chemosensitivity of TNBC cells

**Fig. 5:**
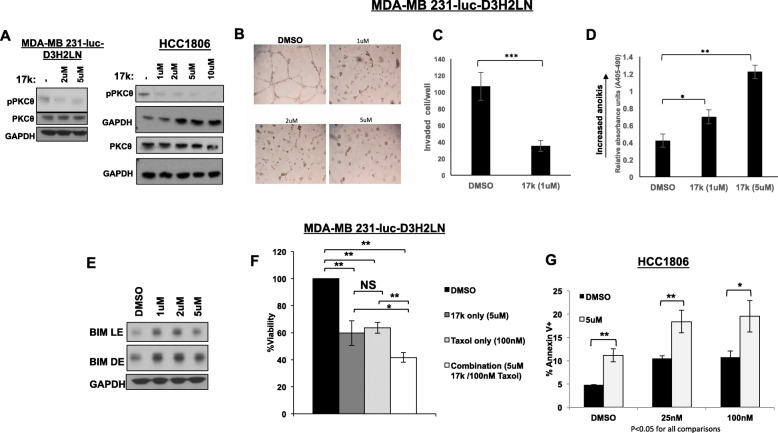
A novel PRKCQ small-molecule kinase inhibitor (17k) suppresses growth and enhances apoptosis of chemotherapy-treated TNBC cells. **a** Treatment of MDA-MB 231-luc or HCC1806 cells with 17k suppresses autophosphorylation of PRKCQ at Threonine 538. Cells were treated at the indicated concentrations for 4 h. 17k treatment suppresses growth in 3D Matrigel culture (**b**), suppresses invasion in Transwell Matrigel assays (**c**), increases anoikis (24 h in suspension cultures) (**d**), and induces Bim expression in suspension cultures (**e**). 17k pre-treatment (48 h) enhances paclitaxel-induced death as measured by the CellTiter-Glo® 3D Luminescence Cell Viability Assay in MDA-MB 231-luc cells (**f**) and by Annexin V+ staining in HCC1806 cells (**g**). For CellTiter-Glo assays, percent viability is plotted relative to DMSO control (set at 100%). All experiments were repeated a minimum of 3 times each. Significance was determined using Student’s *t* test

A novel small-molecule kinase inhibitor of PRKCQ, 17k, was obtained and assessed for its ability to inhibit PRKCQ kinase activity in TNBC cells. 17k is a highly selective inhibitor of PRKCQ, as described in [18], with potent in vitro activity. To determine the effect of 17k inhibitor treatment on endogenous PRKCQ kinase activity in TNBC, we assessed the levels of phosphorylation of Threonine 538 (T538). This is an established autophosphorylation site and is critical for the full activity of PRKCQ [26]. 17k treatment of TNBC cells (MDA-MB231 Luc, HCC 1806) inhibited Thr538 phosphorylation dose-dependently within 4 h of treatment initiation (Fig. 5a). Over longer time periods, 17k treatment also decreased total PRKCQ levels, although levels of other PKC isoforms were not affected (Supplement Figure 1).

After establishing that 17k treatment inhibited endogenous PRKCQ activity in TNBC cells, we determined the effects of inhibitor treatment on oncogenic properties promoted by PRKCQ. 17k treatment inhibited growth and invasive branching of MDA-MB231 cells in 3D Matrigel cultures, suppressed invasion in Matrigel™ Transwell invasion assays, increased anoikis, and induced expression of pro-apoptotic Bim, thus largely phenocopying effects of PRKCQ shRNA (Fig. 5b–e). 17k treatment also suppressed the expression of mesenchymal markers like Vimentin and β5 integrin, suggesting a partial EMT reversal (Supplemental Figure 2). 17k treatment did not suppress the growth of TNBC cells that do not express PRKCQ, supporting the specificity of 17k’s effects (Supplemental Figure 3).

Combined 17k and paclitaxel treatment also suppressed the viability of TNBC cells in 3D culture (as assessed by 3D CellTiter-Glo®) and increased apoptosis (as assessed by levels of Annexin V+ cells) to a greater extent than treatment with either 17k or paclitaxel alone (Fig. 5f, g). These results collectively support the synergistic effects of PRKCQ kinase activity inhibition and chemotherapy in suppressing growth and viability of TNBC cells, and the potential of PRKCQ kinase inhibitors to complement chemotherapy in the treatment of TNBC.

## Discussion

The lack of systemic therapy options for triple-negative breast cancer highlights the need for novel therapeutic strategies. Chemotherapy sensitivity is a consistently strong predictor of outcomes for patients diagnosed with TNBC [1, 2]. However, there is heterogeneity in sensitivity to chemotherapy among TNBC, and it is critical to identify targetable drivers of chemotherapy resistance. PRKCQ is a novel PKC family member, preferentially expressed in the TNBC subtype and drives growth factor-independent growth, anoikis resistance, migration, and EMT, all properties associated with tumorigenesis and metastatic spread of cancer cells [14, 16, 17, 27]. EMT specifically is associated with chemotherapy resistance via multiple mechanisms in several tumor types (reviewed in [28]). Expression of LSD1, a downstream substrate of PRKCQ and driver of EMT, was reported to be enriched among chemotherapy-resistant triple-negative xenograft tumors and patient circulating tumor cells [17]. LSD1, a histone demethylase, is activated during EMT and in turn activates multiple EMT programs to promote chemotherapy resistance. Here, we have identified a novel mechanism by which PRKCQ critically regulates the apoptotic response to chemotherapy treatment, specifically by regulating levels of pro-apoptotic Bim. Our data further support the promising potential of PRKCQ inhibition as a therapeutic strategy to suppress TNBC tumor growth and survival in combination with standard-of-care chemotherapy.

Expression levels and activation of Bcl2 family members critically determine the sensitivity of cancer cells to chemotherapy and targeted therapies (reviewed in [22]). Several PKC isoforms have been shown to promote resistance to chemotherapy by upregulating expression of anti-apoptotic Bcl2 or XIAP. However, our results are the first to show suppression of pro-apoptotic Bim as a mechanism for chemotherapy resistance downstream of PRKCQ, the only PKC isoform preferentially expressed in TNBC [14]. Expression of Bim is required for apoptosis induction by cytotoxic agents, as well as targeted therapies like imatinib, dasatinib, and erlotinib [23]. Bim promotes apoptosis by activating Bax/Bak, and it is regulated by both transcriptional and post-translational mechanisms. Our results support PRKCQ-dependent regulation of Bim is at least partially due to the regulation of its transcript levels.

Small-molecule PRKCQ inhibitors could be a promising therapeutic approach given the kinase dependency of PRKCQ’s oncogenic activities and promotion of chemotherapy resistance. Kinase activity dependency is supported by (1) the failure of catalytically inactive PRKCQ to promote chemotherapy resistance or suppress Bim, and (2) the ability of PRKCQ kinase inhibitor to significantly phenocopy the effects of PRKCQ shRNA with respect to growth inhibition and apoptosis induction. However, kinase-independent functions of PRKCQ are still possible, given the more significant increase in apoptosis observed with TNBC cells co-treated with chemotherapy and PRKCQ shRNA compared to PRKCQ kinase inhibitor.

Several PRKCQ kinase inhibitors, including the one used in our studies, have been developed mostly for the purposes of immunomodulation in the setting of autoimmune diseases or transplant rejection [29]. Our results support an oncogenic role for PRKCQ and support targeting this novel PKC isoform in TNBC. Concerns about the immunological consequences of targeting PRKCQ in non-autoimmune settings may be alleviated by the findings from studies of PRKCQ-null mice that suggest that PRKCQ is largely dispensable for anti-microbial and anti-viral immunity [10]. Moreover, the consequences of PRKCQ inhibition on the tumor immune microenvironment may be favorable, as suggested by the results with an inhibitor of LSD1, a downstream target of PRKCQ [17]; LSD1 inhibition enhanced recruitment of M1 type macrophages that could favorably contribute to anti-tumor immunity.

While the in vitro kinase profiling of the PRKCQ kinase inhibitor used in this study supports its specificity, there were some toxicities observed with related inhibitors in the context of in vivo studies of autoimmunity and inflammation that were not observed in the PRKCQ-null mice [18]. Whether these represent toxicities specific to the models used (non-tumor bearing) or generalizable off-target effects at the indicated doses remains to be determined.

Although the detection of loss-of-function mutations in specific PKC isoforms has raised the possibility that some PKC family members may have tumor-suppressive functions (reviewed in [30]), it is likely that specific isoforms have pro- or anti-tumorigenic roles depending on the tumor tissue of origin and relative expression levels of these isoforms. The growing body of studies demonstrating oncogenic functions of PRKCQ support further genetic and functional analysis of this isoform in the context of TNBC to identify optimal strategies for clinical translation.

## Conclusions

The PRKCQ expression regulates the sensitivity of TNBC cells to the apoptotic effects of chemotherapy treatment by regulating the expression of Bim, a pro-apoptotic Bcl2 molecule. Regulation of Bim and apoptosis in chemotherapy-treated cells is dependent on PRKCQ kinase activity, raising the possibility that PRKCQ kinase inhibitors can be used to complement standard chemotherapy to inhibit TNBC.

## Supplementary information

**Additional file 1 **: **Supplemental Figure 1. PRKCQ kinase inhibitor (17k) treatment does not affect other PKC isoforms.** MDA-MB231Luc cells were treated with indicated concentrations of 17k for 24 hours. Expression of PKC isoforms was assessed. **Supplemental Figure 2. PRKCQ inhibition suppresses expression of mesenchymal proteins.** MDA-MB231Luc cells were treated for 24 hours with indicated concentrations of 17k. Expression of Vimentin and β5 integrin was assessed. **Supplemental Figure 3. PRKCQ inhibitor (17k) treatment does not inhibit growth of TNBC cells that do not express PRKCQ**. MDA-MB157 cells that do not express PRKCQ were cultured in 3D Matrigel cultures and treated for the indicated number of days with 17k (1uM). MDA-MB231Luc cells were plated at the same time in parallel cultures and treated with 17k (1uM) for the same number of days and serves as a positive control for the efficacy of 17k.

## Data Availability

All data generated or analyzed during this study are included in this published article and its supplementary information files.

## Abbreviations

PRKCQ/PKCθ: Protein kinase C theta
EMT: Epithelial-mesenchymal transition
TNBC: Triple-negative breast cancer
