# Supplementary figures and images for: PRKCQ inhibition enhances chemosensitivity of triple-negative breast cancer by regulating Bim
Source: Breast Cancer Res. 2020 Jun 29;22:72. doi: 10.1186/s13058-020-01302-w (PMC7322866; doi:10.1186/s13058-020-01302-w)

Supplementary Fig 1

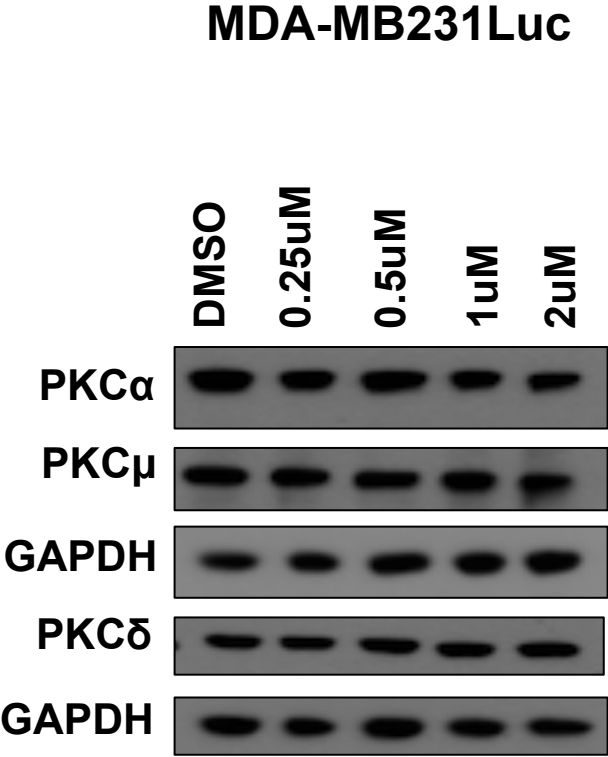

Supplementary Fig 2

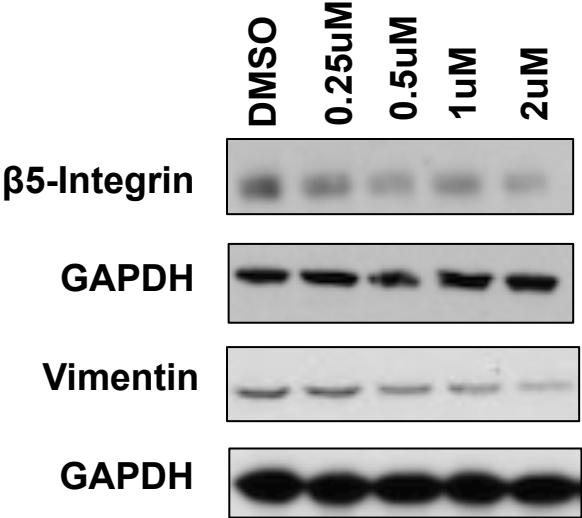

**Supplementary Fig 3**

**Day 4**

**Day 8**

**DMSO**

**1uM 17k**

**DMSO**

**1uM 17k**

**MDA-157**

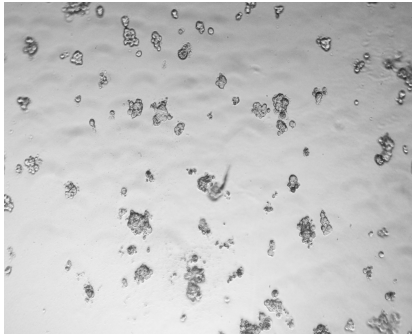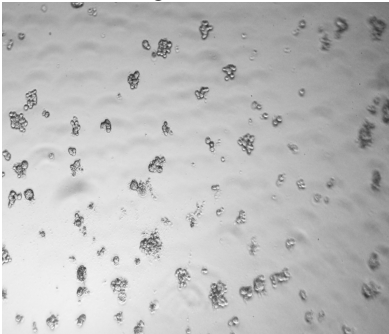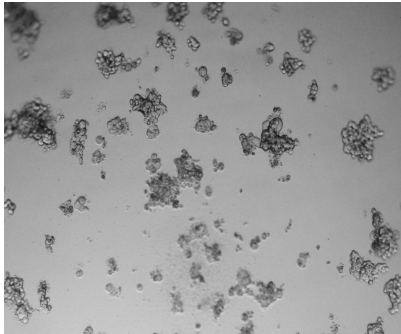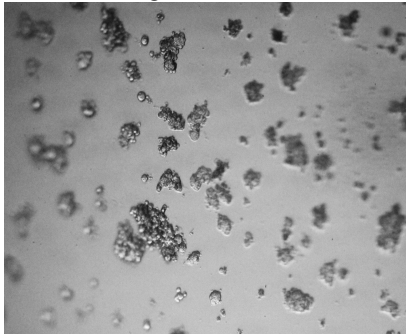

**MDA-MB  
231-Luc**

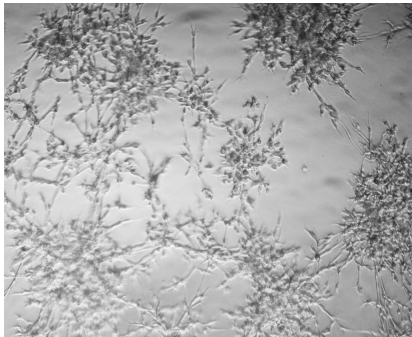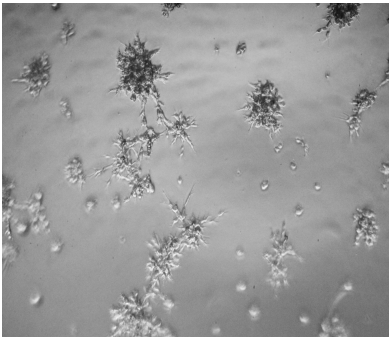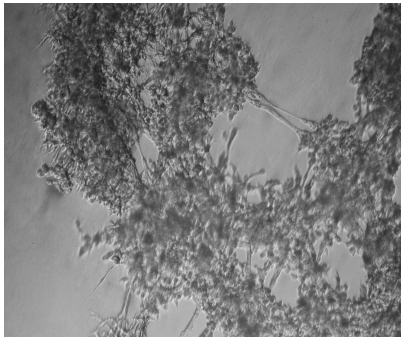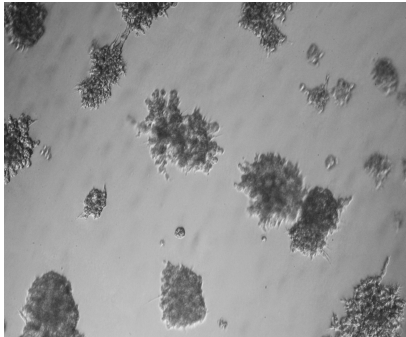

Supplement: Supplementary file 1 — Additional file 1 : Supplemental Figure 1. PRKCQ kinase inhibitor (17k) treatment does not affect other PKC isoforms. MDA-MB231Luc cells were treated with indicated concentrations of 17k for 24 hours. Expression of PKC isoforms was assessed. Supplemental Figure 2. PRKCQ inhibition suppresses expression of mesenchymal proteins. MDA-MB231Luc cells were treated for 24 hours with indicated concentrations of 17k. Expression of Vimentin and β5 integrin was assessed. Supplemental Figure 3. PRKCQ inhibitor (17k) treatment does not inhibit growth of TNBC cells that do not express PRKCQ. MDA-MB157 cells that do not express PRKCQ were cultured in 3D Matrigel cultures and treated for the indicated number of days with 17k (1uM). MDA-MB231Luc cells were plated at the same time in parallel cultures and treated with 17k (1uM) for the same number of days and serves as a positive control for the efficacy of 17k. [file 13058_2020_1302_MOESM1_ESM.pdf]
